# Effect of temperature on yellow leaf disease symptoms and its associated areca palm velarivirus 1 titer in areca palm (*Areca catechu* L.)

**DOI:** 10.3389/fpls.2022.1023386

**Published:** 2022-10-14

**Authors:** Latif Ullah Khan, Xianmei Cao, Ruibai Zhao, Hang Tan, Zengyu Xing, Xi Huang

**Affiliations:** Sanya Nanfan Research Institute, Hainan University, Sanya, China

**Keywords:** Areca palm, areca palm velarivirus 1 (APV1), yellow leaf disease (YLD), seasonal effects, symptoms severity, temperature

## Abstract

Yellow leaf disease (YLD) has been a major limiting factor threatening areca palm commonly known as betel palm (*Areca catechu* L.) plantations in Hainan, China. The YLD disease is closely associated with areca palm velarivirus 1 (APV1), which belongs to the family *Closteroviridae*. YLD-affected betel palms show more serious yellowing symptoms in winter than in summer based on anecdotal observations. In the present work, the underlying mechanism was investigated. We first observed that the severity of YLD symptoms was closely related with the APV1 viral titer determined by qRT-PCR and ELISA under natural conditions. To further investigate whether temperature plays a key role in APV1 accumulation, the areca palm seedlings were artificially inoculated with APV1-positive mealybugs (*Ferrisia virgata*) and then cultivated under controlled conditions. According to our results, the YLD symptoms severity in inoculated seedlings were closely associated with temperature, e.g., severest symptoms at low temperature (16/22 ± 2°C, night/day), severer symptoms at room temperature (24/26 ± 2°C, night/day), while moderate symptoms at high temperature (27/34 ± 2°C, night/day). The qRT-PCR and ELISA results showed that APV1 titer accumulates significantly abundant at low temperature as compared to high and room temperatures. In conclusion, this is the first report about the temperature effects on the symptoms severity of YLD and APV1 titer, which may have important implications for the epidemiology of YLD.

## Graphical Abstract

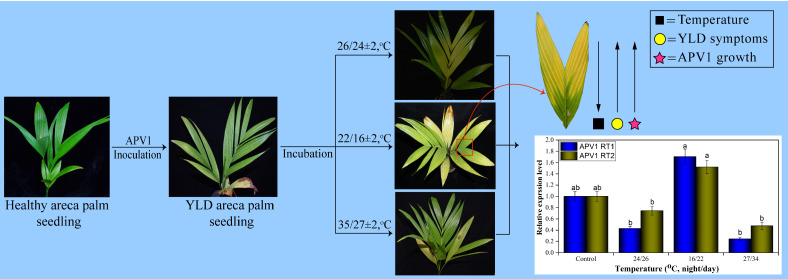

## Introduction

The most grievous threat to areca palm plantations worldwide is yellow leaf disease (YLD), the disease that affects the economy of areca palm up to a high extent in different countries by destroying a large number of areca palm plantations in China (40,000 ha) (Yu et al., 1986; Wang et al., 2020), Sri Lanka (11,968 ha) (Kanatiwela de Silva et al., 2015), and India (about 10,000 ha) (Rawther, 1976; Purushothama et al., 2007). The YLD first emerged in India (Raghavan and Baruah, 1958; Rawther, 1976; Nair et al., 2016); after that, the disease was also examined in Hainan, China (Yu et al., 1986; Wang et al., 2020) and Sri Lanka (Kanatiwela de Silva et al., 2015). Areca palm widely grows in the Pacific, tropical areas of Asia, and East Africa despite the belief that its origin is in the Philippines and Malaysia (Kurian and Peter, 2007). The commercial importance of areca palm is greater in China and India than in other countries (Hattori et al., 1993; Daquan et al., 2001). Religious and masticatory purposes make their significance more unique (Kurian and Peter, 2007). The areca palm plantation is under attack by different pathogens, including bacteria (Kumar, 1983; Nair et al., 2016), fungi (To-anun et al., 2009; Wang et al., 2019), insects such as *Tirathaba rufivena* (Zhong et al., 2017) and *Brontispa longissima* (Li et al., 2007), phytoplasmas (Ramaswamy et al., 2013; Kanatiwela de Silva et al., 2015), and viruses (Yu et al., 2015; Yang et al., 2018a; Yang et al., 2018b; Wang et al., 2020; Cao et al., 2021; Zhang et al., 2022). The approved causal agent of YLD specifically in Hainan, China is the novel virus named areca palm velarivirus 1 (APV1) based on *de novo* assembly, RNA-seq, and next-generation sequencing (Yu et al., 2015; Wang et al., 2020; Cao et al., 2021), while the responsible transmission vectors of APV1 in Hainan are mealybugs *Pseudococcus cryptus* and *Ferrisia virgata* on the basis of immunocapture RT-PCR and immunofluorescence assays (Zhang et al., 2022). The most conspicuous symptom of YLD is the yellowing of the leaves; the symptoms emergence that was observed and recorded in the present research work was the same as those described by Wang et al. (2020) and Zhang et al. (2022), although the severity of the symptoms towards different temperatures and seasonal effects was not known exactly before, and was needed to be explored. The YLD symptoms start with the yellowing of leaflet tips of the lowermost leaves or the mid-crown, or the lowest leaves. At the early stage, a clear demarcation of affected yellow and normal green areas was observed on the infected leaves, which differentiates YLD yellowing from physiological yellowing. Chlorosis spread along the direction of vascular tissue while the midribs remain green, forming a green–yellow border. At the later stage, the yellowing expands to younger and lower leaves, while the green–yellow border on older leaf becomes unclear. Newly emerging leaves are stunted, and the crown size is markedly reduced, resulting in the “bunchy top” symptom (Wang et al., 2020; Zhang et al., 2022). Later, the leaf becomes dark brown, the conductive filaments get demolished, the stem becomes spongy and friable, and finally breaks at the top, the roots rot, the size of the nuts is reduced, and the kernel turns into black and becomes unusable (Rawther, 1976; Zhang et al., 2022). The climate and weather intensely influenced the plant viruses and their vectors at different stages during their lives. It is expected that the establishment, spread, and reproduction potential of plant viruses are greatly affected by climate changes (Kido et al., 2008). Previously, it has been observed anecdotally that under natural environmental conditions, the areca palm YLD symptoms severity in cold and drought stresses becomes more dominant as compared to warm and rainy weather such that the whole areca palm orchards seem to be burnt (Wang et al., 2020; Zhang et al., 2022); thus, in context with the above observations, this is the first report that scrutinizes the YLD symptoms severity and the virus APV1 titer accumulation during different seasons, and explores the role of temperature on YLD intensity. Therefore, the current study was conducted to find out the underlying mechanism and to quantify virus titer both in the field and in the areca palm YLD seedlings maintained under different temperatures in the laboratory under artificially controlled environmental conditions. It was determined that APV1 is closely associated with the areca palm YLD incidence *via* quantitative real-time (qRT)-PCR and enzyme-linked immunosorbent assay (ELISA), and that the temperature plays a key role in YLD intensity. A quantitative assessment of virus titer in plants exposed to different temperature conditions would provide information about the possible effects of future climates on the APV1 infection incidence and YLD intensity; further studies are required to find out the YLD disease control management strategies in order to protect areca palm plantations from the YLD disease incidence.

## Materials and methods

### Plant materials

#### Inoculation of APV1

APV1 was inoculated by mealybugs (*F. virgata*), the transmission vectors of APV1 (Zhang et al., 2022). The mealybugs were fed pumpkin inside a nylon net cage (75 × 75 × 75 cm) at room temperature. First instar mealybugs were used for transmission purposes because they possess more transmission capabilities as compared to adult mealybugs, which may be due to the strong immunity of adult mealybugs (Zhang et al., 2022). After feeding, the mealybugs were transferred to the YLD tree for 48 h of AAP (acquisition access period). After 48 h of AAP, 50 mealybugs carrying APV1 per plant were then transferred for IAP (inoculation access period, 48 h) and enclosed by a micro-perforated tinfoil cage (mesh size ca. 0.2 mm) onto the newly opened leaves of recipient healthy areca palm seedlings in a separate nylon net cage (75 × 75 × 75 cm) in a chamber to protect it from insect contamination. The tinfoil cages were removed after 48 h of IAP, and the acetamiprid, an insecticidal spray, was used to clean the plants from mealybugs. The newly inoculated palms were then kept in a laboratory for at least 1 week to confirm the removal of mealybugs and were then transferred to a chamber until YLD symptoms emerged clearly.

#### Plant sampling and symptoms recording in the field

The YLD plants showing major infection symptoms were used for sample collection and symptoms recording in the field at Hainan, in three different seasons, autumn (September), winter (December), and summer (May). The average daily high- and low-temperature fluctuation map for the whole year of Hainan was obtained from Hainan Weather Forecast Centre. The map showed the daily average maximum and minimum temperatures (°C) during each month. The samples were collected in independent biological triplicates, and all leaves from one plant were considered as one sample. The samples were stored at −80°C until used for further analysis.

#### Plant sampling and symptoms recording under controlled conditions

Two-year-old healthy areca palm seedlings were purchased from the Coconut Institute at Wenchang, China, and were cultured in nylon net cages (75 × 75 × 75 cm) in a chamber (26 ± 2°, 16L:8D, relative humidity [RH] = 75 ± 2%). Ten seedlings for each specific temperature treatment, i.e., room, low, and high, were inoculated with APV1. Healthy areca palm seedlings served as a negative control, while APV1-positive seedlings at room temperature were used as a positive control. When the seedlings were tested positive for APV1 *via* RT-PCR and YLD symptoms emerged clearly after 75 dpi (days post-inoculation) (Zhang et al., 2022), they were kept in an incubator under controlled growth conditions having artificial light at low temperature (16/22 ± 2°C, night/day, 16L:8D), high temperature (27/34 ± 2°C, night/day, 16L:8D), and room temperature (24/26 ± 2°C, night/day, 16L:8D). The time period used in the current study for each incubation was 1 month, and after that, the symptoms were recorded thoroughly, and the leaf samples were stored at −80°C, until used for further analysis.

#### Total RNA extraction

Total RNA was extracted from each sample by using the Universal Plant Total RNA Extraction Kit (BioTeke, China) following the manufacturer’s instructions. The quality and purity of the extracted RNA were examined using agarose gel electrophoresis, and a NanoPhotometer^®^ spectrophotometer (Implen, CA, USA).

#### cDNA synthesis and qRT-PCR analysis

The extracted total RNA was further used for the synthesis of cDNA through RT-PCR by using TransScript^®^ One-Step gDNA Removal and cDNA Synthesis SuperMix (TransGen Biotech, China) kit with random primers, following the manufacturer’s instructions. After the second strand was synthesized successfully, the cDNA was used as a template for qRT-PCR analysis. The qRT-PCR analysis was performed on a Light cycler 96/Light cycler 480 Real-time System (Roche diagnostic, UK) using the TB Green Premix Ex Taq II (Tli RNaseH Plus) (2X) (TaKaRa) kit following the manufacturer’s instructions. In detail, each RT reaction contained 2 μl of diluted cDNA (1:10), 10 μl of the TB Green Premix Ex Taq II (Tli RNaseH Plus) (2X) PCR mixture, 0.4 μl of each 10 mM primer, and RNase-free ddH_2_O to a final volume of 20 μl. The qRT-PCR program conditions were as follows: Pre-incubation, 95°C for 30 s; followed by 40 cycles of amplification, 95°C for 5 s, 60°C for 15 s, and 72°C for 10 s; followed by melting, 1 cycle of 95°C for 10 s, 65°C for 60 s, and 97°C for 1 s; and final cooling, 37°C for 30 s. All the qRT-PCR analyses were performed in biological triplicates. For APV1 gene expression, two primers were used in the current study: APV1-RT1, which encodes a 160-bp product in ORF9, and APV1-RT2, which encodes a 154-bp product in ORF7 of the APV1 genome. Primer3 software was used for primer design. PDS was used as the internal control. The list of primers used in the present study is given in **Table 1**. The relative expression levels of the genes were calculated by a comparative CT method (ΔΔCT) using the 2^−ΔΔCt^ method (Livak and Schmittgen, 2001).

**Table 1:** List of primers used in the current study.

Primer number	Primer name	Sequence (5`-3`)	Primer size (bp)	Product size (bp)
1	AcPDS-F	GGCTAAACTCTCCTGGCTTG	20	100
2	AcPDS-R	CTGCAGAACTTGTTTGGGGA	20	
3	APV1-RT1-F	ACGCACACCAACTAATGATCAAGA	24	160
4	APV1-RT1-R	TAGAGTCATGGGAACTCGCAAATT	24	
5	APV1-RT2-F	CATCGGTTGTTATGACCATACCAA	24	154
6	APV1-RT2-R	TTAAGAAGACTTCTTGGAGTGCCT	24	

#### Enzyme-linked immunosorbent assay

DAC-ELISA was developed for the quantification of APV1 titer in YLD areca palm by following the standard protocol of Koenig (1981), with slight changes. The YLD areca palms at different temperatures were taken as a test antigen, the negative control was the leaves of healthy seedlings, and the positive control was the APV1-positive plant at room temperature. The antigen was extracted by taking an appropriate amount of the samples and was ground with liquid nitrogen into a fine powder and was quickly loaded in a 2-ml centrifuge tube; the sample was weighed and the coating buffer i.e 0.05 M carbonate buffer, (Na_2_CO_3_ 0.159g; NaHCO_3_ 0.294g, pH 9.6) was added at a ratio of 1:10 (W/V). The homogenate was mixed thoroughly and centrifuged at 12,000 rpm at 4°C for 10 min. The 96-well microliter plate (Nest Scientific, USA) was first coated with antigen (100 μl/well) and was incubated at 4°C, overnight. After washing with PBS-T and blocking with 2.5% skimmed milk (200 μl/hole), the primary antibody (100 μl/well) APV1 serum was diluted at a ratio of 1:300 in 1× PBS-T buffer, was added to each well, and was then incubated at 37°C for 2 h. After washing with PBS-T, alkaline phosphatase-labeled goat anti-mouse IgG secondary antibody (100 μl/well) diluted with 1× PBS-T buffer at a ratio of 1:10,000 was added to each well and incubated at 37°C for 2 h. After that, the freshly prepared substrate tetra-methyl benzidine (TMB) solution (100 μl) was added to each well and was kept in the dark for 20 or 30 min at 37°C until the color developed to the desired level. The reaction was stopped by adding a stopping solution of 1 M concentrated HCl (50 μl/well), and the reading was taken at OD 450 nm (BioTek Synergy H1, Microplate Reader, USA). Results were calculated according to the formula [cutoff value (P/N) = (sample OD − blank OD)/(negative OD − blank OD)]. A mean absorption value of the tested samples twice higher than that of healthy plant antigen was taken as positive, indicative of disease status (Sutula et al., 1986).

## Results

### YLD symptoms severity in different seasons in the field

**Figure 1:**
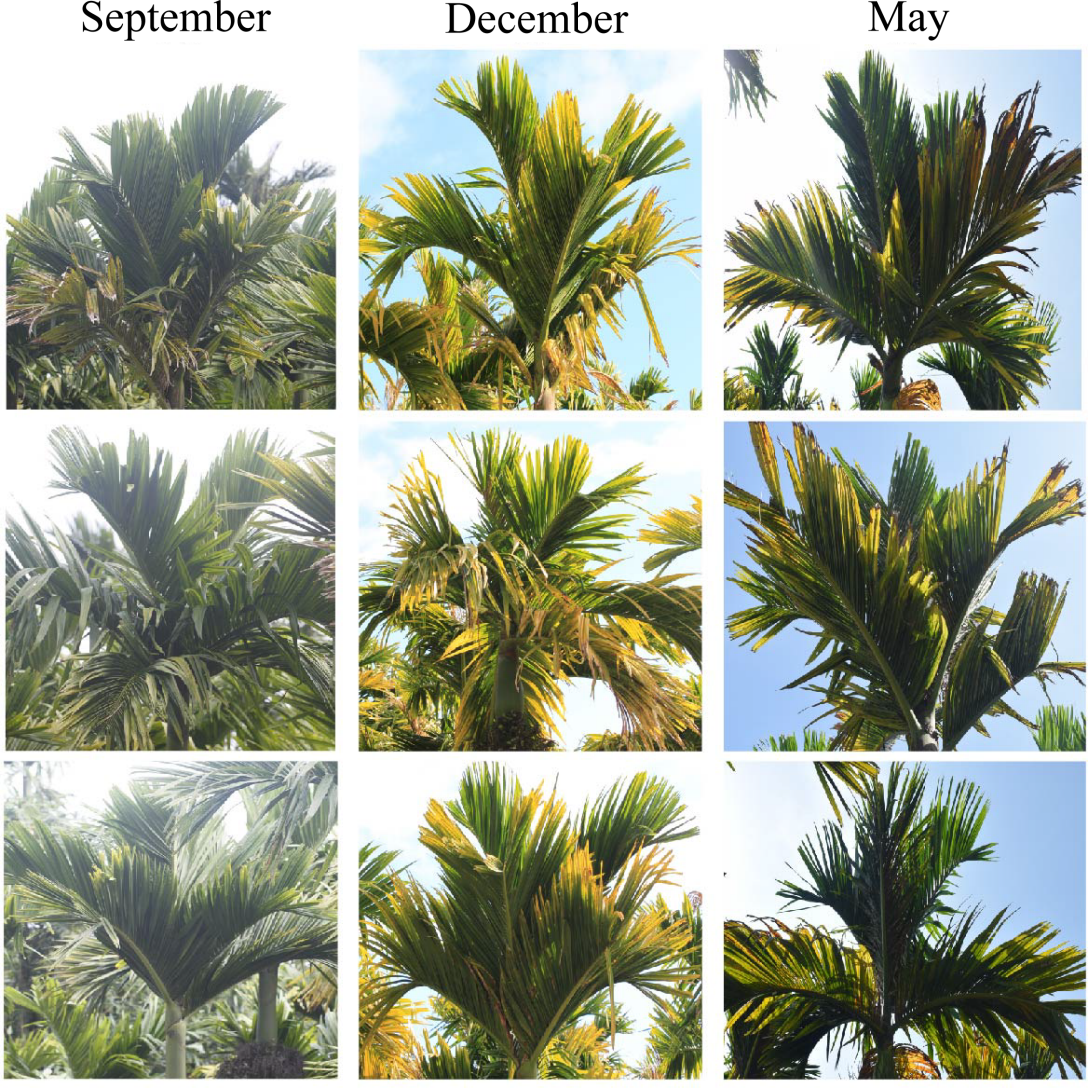
Symptoms of YLD recorded in different seasons in the field. (September) The YLD symptoms that were recorded in the autumn season (moderate temperature and rainy weather). (December) The symptoms severity recorded in the winter (low temperature and dry weather).(May) The YLD symptoms recorded in the summer (high temperature and warm weather). The symptom’s severity was markedly higher in winter than in autumn and summer.

**Figure 2:**
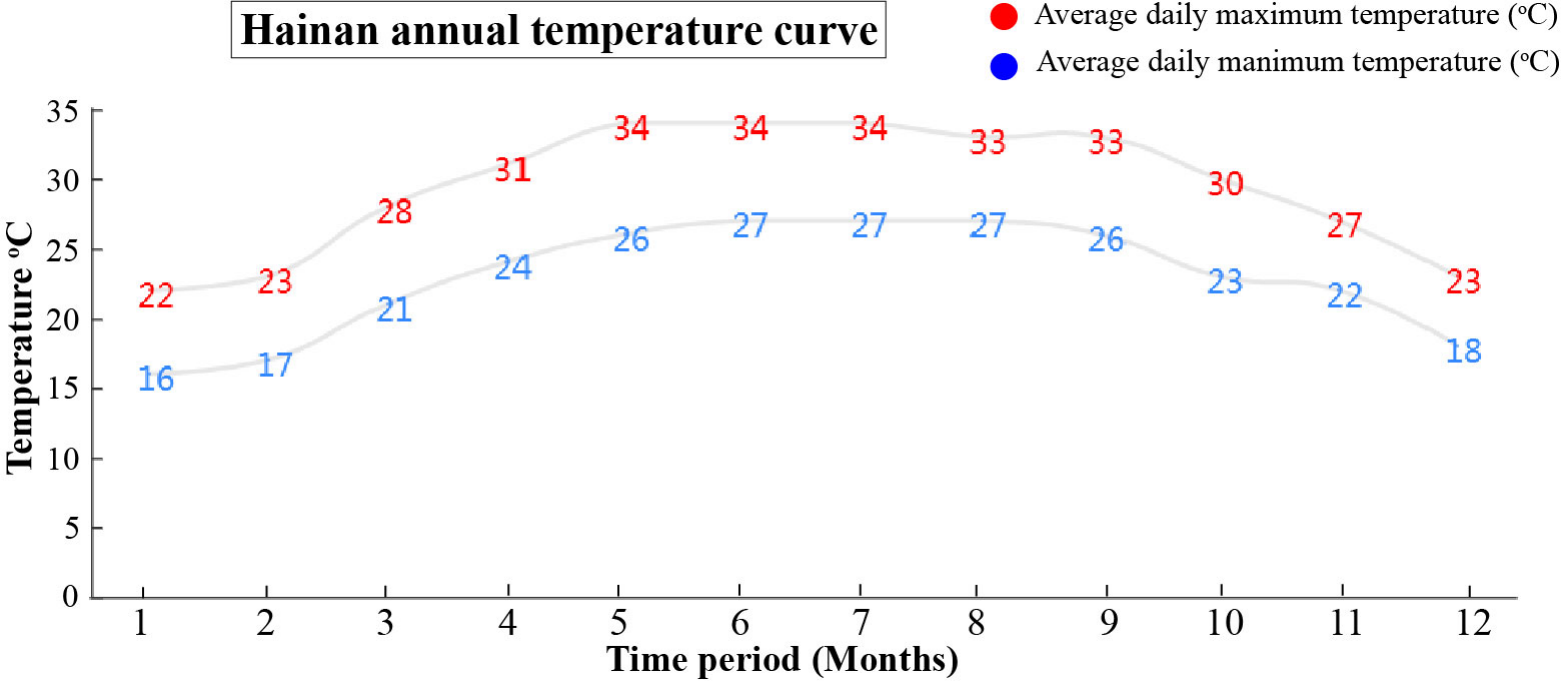
The graph showed the daily average high (red) and low (blue) temperatures of Hainan, used in the current study. The numbers of the time period from 1 to 12 expressed the months of data collection year.

The basic symptoms of YLD were the same as observed previously but little was known about the effects of different seasons on YLD intensity. Thus, for the confirmation of seasonal effects, we record the symptoms and collect the samples of YLD areca palms in disease pandemic areas during various seasons throughout the year under natural conditions in the field, as shown in **Figure 1**, in which “September” indicated the emergence of symptoms of YLD during the autumn season, in rainy weather; “December” showed the symptoms of YLD that appeared in winter, in cold weather; and “May” indicated the symptoms of YLD in summer, in warm weather. These results clearly showed that YLD symptoms severity is evidently higher in winter, under cold weather conditions (December), and the leaves become more seriously yellowish as compared to rainy and warm seasons (September and May, respectively). The close observation of YLD symptoms emergence throughout the year (**Figure 1**) revealed that in summer and in the rainy season, the disease symptoms gradually become milder and the leaves turned green up to a high extent, but at the end of the year (December) and at the beginning of the year (January and February), i.e., in winter, the disease symptoms became severest and the leaf yellowness becomes remarkably high, and the whole areca palm plantation appears to be burnt, which means that cold stress has a greater effect on YLD intensity. However, we cannot totally conclude that temperature plays a key role in disease intensity because under natural conditions, other factors also exist, such as humidity and insects; thus, it is essential to specifically investigate the role of temperature on disease intensity under controlled conditions. The average daily maximum and minimum temperatures of Hainan are shown in **Figure 2**, which was obtained from the Hainan Weather Forecast Centre.

### qRT-PCR and ELISA for analysis of APV1 titer accumulation in different seasons

**Figure 3:**
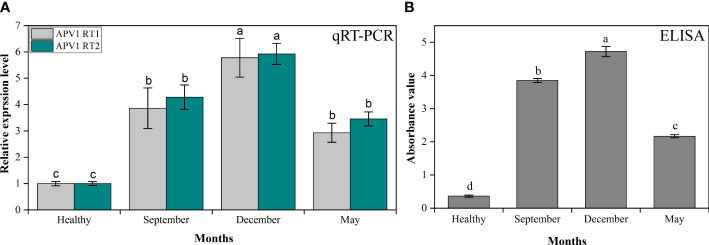
The APV1 titer accumulation analysis by **(A)** qRT-PCR in control (healthy), autumn (September), winter (December), and summer (May) seasons. The primer APV1-RT1 that encodes APV1-ORF9, and APV1-RT2 that encodes APV1-ORF7 were used for gene expression analysis. **(B)** The APV1 titer was detected by ELISA using a polyclonal antibody against APV1 coat protein. The absorbance values of APV1-positive seedlings in different seasons under natural environmental conditions were taken at OD 450 nm using a microplate reader (BioTek Synergy H1, USA). The results were calculated according to the formula [cutoff value (P/N) = (sample OD − blank OD)/(negative OD − blank OD)]. A mean absorption value of the tested samples twice higher than that of healthy plant antigen was taken as positive for the disease. Positive controls are the mean of absorbance readings of wells containing APV1-infected areca palm leaf samples, while negative controls are the mean of absorbance readings of healthy areca palm leaves. Data presented as means ± standard error, *n* = 9; significant differences are exhibited by lowercase letters (*p* ≤ 0.05), according to the LSD test.

The YLD symptoms severity observed in the present study was remarkably higher in winter than in summer, and was closely associated with APV1 through qRT-PCR and ELISA. For the analysis of APV1 gene accumulation, we have used two primers, i.e., APV1-RT1 and APV1-RT2, that encode APV1 ORF9 and ORF7, respectively, and PDS was used as a control for normalization of the samples (**Table 1**). The results revealed that under cold and dry environmental conditions (winter), APV1-RT1 expressed abundantly (5.78 ± 0.736-fold) as compared to control (healthy), while it expressed 3.87 ± 0.771-fold in the rainy season (September) and 2.93 ± 0.361-fold in the warm season (May), which means that APV1 gene overexpressed in cold weather (5.78-fold) as compared to control, 1.49-fold higher as compared to the rainy condition, and 2-fold greater as compared to the warm condition, while APV1-RT2 also accumulates more (5.93 ± 0.401-fold) as compared to control (healthy) in winter, while it expressed 4.29 ± 0.462-fold in September and 3.46 ± 0.267-fold in May. Under cold conditions, APV1-RT2 expressed highly, 5.93-fold, 1.38-fold, and 1.72-fold as compared to control (healthy), rainy, and warm weather conditions, respectively, as shown in **Figure 3A**. ELISA was also adapted for the efficient detection of APV1 titer accumulation during different seasons in the field under natural conditions. The findings showed that YLD areca palms during cold and dry seasons, i.e., December, have higher APV1 titer accumulation (4.722 ± 0.155), i.e., 13.04 times higher as compared to healthy areca palms (0.362 ± 0.035), while titer abundance in rainy weather, i.e., September (3.847 ± 0.060), and in hot weather, i.e., May (2.168 ± 0.048), was recorded. It means that APV1 under cold conditions expressed 1.22 times and 2.18 times more as compared to rainy and hot weather, respectively (**Figure 3B**). These results clearly indicated that APV1 titer accumulation in winter is remarkably higher than that in summer.

### Transmission of APV1 *via* mealybugs/inoculation of APV1

**Figure 4:**
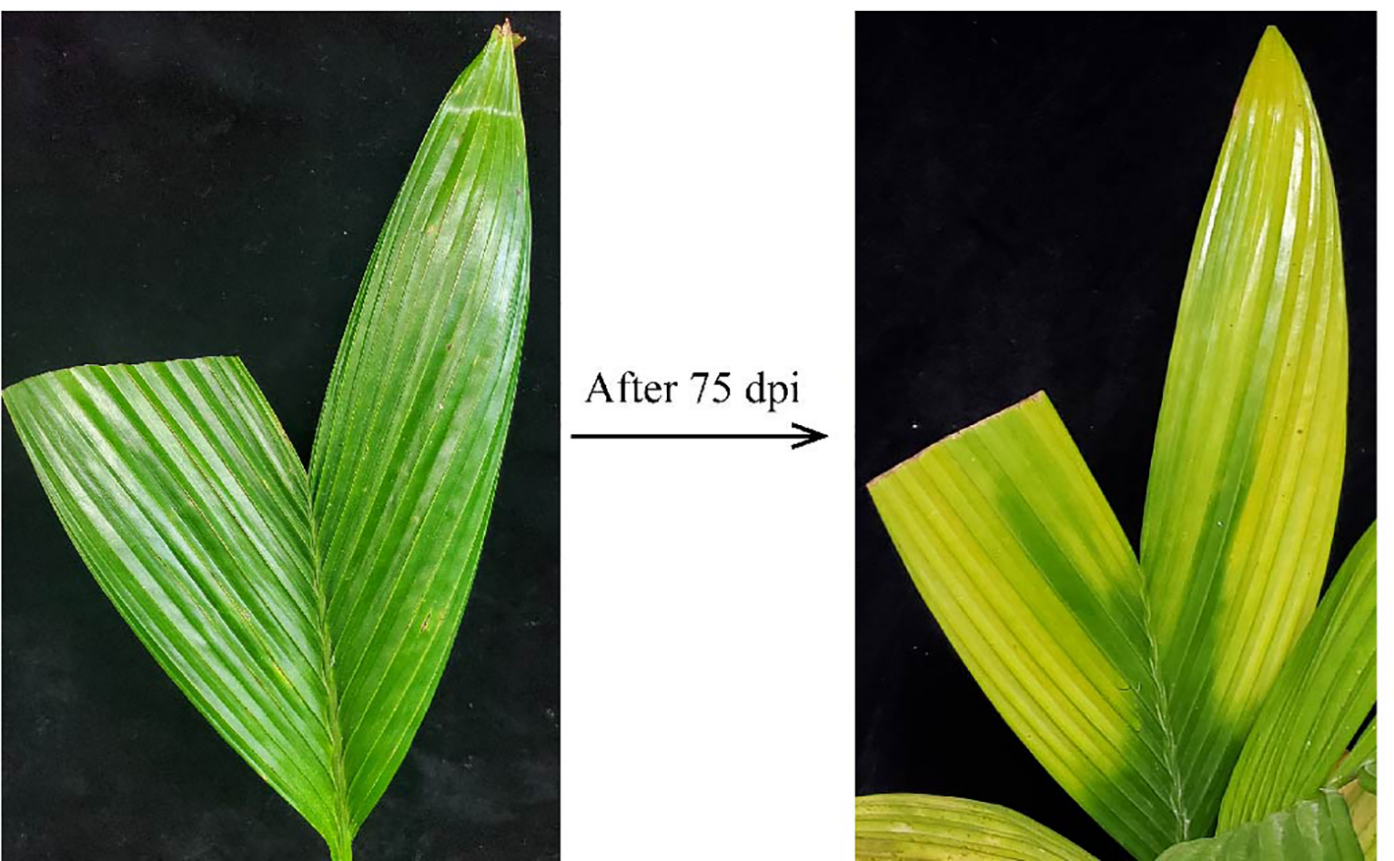
APV1 YLD symptoms emerged after 75 days post-inoculation (dpi).

The mealybugs *F*. *virgata* were used for the transmission of APV1, as described in the previous investigation by Zhang et al. (2022), and both the AAP and IAP of APV1 were recorded for 48 h. After 60 dpi, the seedlings have become APV1 positive, and after 75 dpi, the YLD symptoms have clearly emerged. Chlorosis begins at leaflet tips and spread in the direction of vascular tissue. The midribs remained green, and yellow–green borders were formed, which distinguished them from physiological yellowing (**Figure 4**). The YLD symptoms observed and recorded in the current study were the same as those described by Zhang et al. (2022) and Wang et al. (2020), and strongly agreed with the results of Zhang et al. (2022) in which APV1 is transmitted by mealybugs and caused YLD on areca palm seedlings.

### YLD symptoms severity at different temperatures under controlled conditions

**Figure 5:**
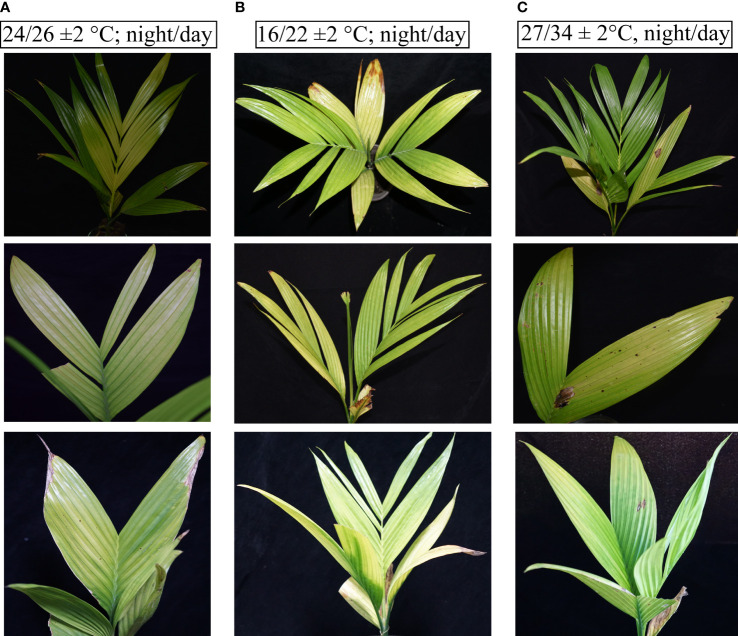
Symptoms of YLD recorded under different temperatures. **(A)** The YLD symptoms emerged at room temperature (24/26°C; night/day). **(B)** The YLD symptoms emerged at low temperatures (16/22 ± 2°C; night/day, 16L:8D; Light, 5LS; RH, 75%). **(C)** The YLD symptoms emerged at high temperatures (27/34 ± 2°C, night/day, 16L:8D; Light, 5LS; RH, 75%). Ten seedlings for each specific temperature analysis, *n* = 10, were kept in an incubator under controlled conditions for 1 month.

The different seasonal effects on YLD symptoms severity reveal that the symptoms become severest in winter than in summer, as described earlier, but it was not sure that these symptoms’ severeness was due to temperature because under natural environmental conditions, other factors exist too; thus, for confirmation that either temperature plays any role in YLD intensity or not, the same experiment was also conducted in the laboratory under controlled conditions in a chamber. The healthy areca palm seedlings were artificially inoculated with APV1, and after 75 dpi, the symptoms emerged clearly (**Figure 4**). After the emergence of the symptoms and confirmation of the disease through RT-PCR, 10 seedlings per treatment were kept in an incubator under controlled growth conditions having artificial light, 27/34 ± 2°C, night/day, 16L:8D; Light, 5LS; RH, 75 ± 2%, for high temperature, and 16/22 ± 2°C, night/day, 16L:8D; Light, 5LS; RH, 75 ± 2% for low temperature, and 10 seedlings were also kept at room temperature. All specific temperatures were obtained from Hainan Weather Forecasting Center. The symptoms that emerged in response to different temperatures are shown in **Figure 5**. The symptoms clearly showed that at low temperatures (**Figure 5B**), the YLD symptoms were markedly dominant as compared to room temperature (**Figure 5A**) and high temperature (**Figure 5C**), which means that temperature plays a key role in YLD symptoms severity, because during controlled conditions, temperature was the only variable parameter in high- and low-temperature treatments. Thus, it was concluded that temperature plays a key role in YLD symptoms severity.

### qRT-PCR and ELISA for analysis of APV1 titer accumulation under different temperatures in the laboratory

**Figure 6:**
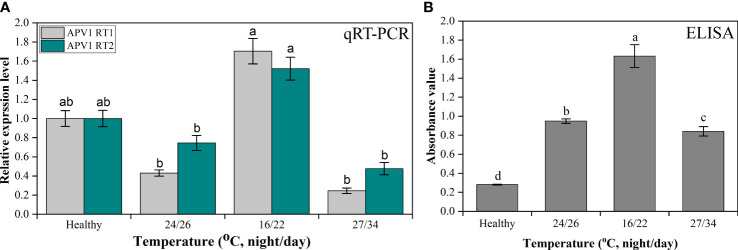
The APV1 titer accumulation analysis through **(A)** qRT-PCR in control (healthy), room temperature (24/26°C, night/day), low temperature (16/22°C, night/day), and high temperature (27/34°C, night/day). The primer APV1-RT1 that encodes APV1-ORF9, and APV1-RT2 that encodes APV1-ORF7 were used for gene expression analysis. **(B)** The APV1 titer was determined by ELISA using a polyclonal antibody against APV1 coat protein. The absorbance values of APV1-positive seedlings in different seasons under natural environmental conditions were taken at OD 450 nm using a microplate reader (BioTek Synergy H1, USA). The results were calculated according to the formula [cutoff value (P/N) = (sample OD − blank OD)/(negative OD − blank OD)]. A mean absorption value of the tested samples twice higher than that of healthy plant antigen was taken as positive for the disease. Positive controls are the mean of absorbance readings of wells containing APV1-infected areca palm leaf samples, while negative controls are the mean of absorbance readings of healthy areca palm leaves. Data presented as means ± standard error, *n* = 9; significant differences are exhibited by lowercase letters (*p* ≤ 0.05), according to the LSD test.

After investigation and confirmation of APV1 titer accumulation in the field during different seasons, it was also important to investigate the role of temperature on YLD disease intensity; thus, for this reason, the APV1 titer was also analyzed under different temperatures, i.e., room, low, and high, in the laboratory under controlled growth conditions. The outcomes showed that, at low temperature, the APV1-RT1 expressed higher (1.704 ± 0.132-fold) as compared to control (healthy), while the virus accumulation decreased at room (0.43 ± 0.032-fold) and at high temperature (0.245 ± 0.028-fold), respectively. qRT-PCR results showed that APV1-RT1 expressed 3.96-fold higher than at room temperature, 6.955-fold more as compared to high temperature, while the APV1-RT2 also expressed greatly (1.521 ± 0.119-fold) as compared to control (healthy) at low temperature, while it expressed 0.746 ± 0.078-fold at room temperature and 0.476 ± 0.063-fold at high temperature. At low temperature, APV1-RT2 expressed 2.04-fold and 3.19-fold greater as compared to room and high temperature, respectively, as shown in **Figure 6**. The ELISA results of YLD areca palm seedlings maintained at different temperatures in a controlled growth chamber showed that APV1 titer accumulation was greater at low-temperature conditions (1.632 ± 0.12), which means 5.73 times higher as compared to control (healthy) (0.2805 ± 0.005), while it accumulates 1.72 times and 1.943 times more as compared to YLD seedlings maintained at room (0.949 ± 0.023) and high (0.841 ± 0.05) temperatures, respectively (**Figure 6**), which validates the results of qRT-PCR under control laboratory conditions. These results clearly indicated that APV1 titer was abundantly high at low temperature as compared to room and high temperatures, and showed that temperature is the key factor that plays a role in YLD intensity.

## Discussion

In 1914, the symptoms of YLD of areca palm were observed for the first time in India. However, the etiological ambiguity existed for such a long time (Raghavan and Baruah, 1958; Rawther, 1976); later on, in 1985, the disease was also examined in Hainan, China, and it was declared that this is caused by potassium deficiency (Yu et al., 1986; Wang et al., 2020). The disease was also observed in Sri Lanka (Kanatiwela de Silva et al., 2015). Based on 16S rRNA gene PCR amplification and electron microscopy observations, the disease was linked to phytoplasma in India (Nayar and Seliskar, 1978; Manimekalai et al., 2010; Ramaswamy et al., 2013), China (Daquan et al., 2001; Che et al., 2010; Yu et al., 2020), and Sri Lanka (Kanatiwela de Silva et al., 2015; Abeysinghe et al., 2016), although there was no etiological evidence (Purushothama et al., 2007). According to Wang et al. (2020), they could not find any phytoplasma in YLD-infected plantations from different epidemic areas in Hainan. However, based on RNA-seq (Yu et al., 2015), next-generation sequencing, immuno-capture RT-PCR and immunofluorescence assays (Zhang et al., 2021), and RNA-seq and *de novo* assembly (Cao et al., 2021), it was concluded that APV1 is the causal agent of YLD in Hainan, China. The APV1 belongs to the family *Closteroviridae*, having a flexuous and filamentous shape observed under transmission electron microscopy (TEM), the typical morphology of species in the family *Closteroviridae* (Wang et al., 2020). The genome of APV1-HN associated with YLD from Hainan, China was first sequenced through RNA-seq and was 16,080 nt in length (Yu et al., 2015), while the complete genome sequence of APV1-WYN (Wanning) isolate was approximately 1.5 kb longer than the APV1-HN genome and was recorded to be 17,546 nt in length. In our previously published data regarding APV1 genetic diversity throughout Hainan (China), 20 new complete genomes of APV1 isolates have been identified and sequenced through RNA-seq and *de novo* assembly and were divided into three phylogroups based on phylogenetic analysis, in which phylogroup A is the most prominent group (>70%), which consists of 16 isolates (Cao et al., 2021). The majority of plant viruses are utterly dependent on vectors for transmission purposes; among these vectors, arthropods, mostly Hemipterans, are most commonly used by plant viruses (Ng and Falk, 2006). During the life cycle of a virus, the transmission by vectors is an important stage. The characteristics and mechanism of plant virus transmission by Hemipteran vectors are defined by many terms and parameters, including retention time, acquisition time, latent period, trans-stadial passage, replication, and transovarial transmission ways. The transmission modes were divided into four categories based on their distinct properties: persistent-propagative, persistent-circulative, semi-persistent, and non-persistent (Nault, 1997; Ng and Falk, 2006). It has been investigated in our previous study that APV1 is transmitted in a semi-persistent, non-circulative manner by its transmission vectors *F. virgata* and *P. cryptus* mealybugs (Zhang et al., 2021). The vectors of APV1 were ascertained using immuno-capture RT-PCR and immunofluorescence assays. Along with these, the AAP, IAP, symptoms emergence duration, retention time, and accumulation sites of APV1 inside the vector were also investigated in our previous study. The basic symptoms of YLD observed in the current study were the same as described previously (Wang et al., 2020; Zhang et al., 2022) (**Figure 5**), while very little was known about the effects of climate change on YLD symptoms severity and APV1 titer accumulation. Ours is the first report regarding different temperature effects on YLD intensity. In the present research work, the YLD symptoms observation under natural environmental conditions in the field showed that YLD symptoms severity was markedly higher in winter as compared to summer, and was closely associated with APV1 through qRT-PCR and ELISA. The APV1 titer was evidently higher in winter than in summer. Thus, to further validate that either temperature plays any role in YLD disease intensity or not, the same experiment was also conducted in the laboratory under different temperatures in an artificially controlled conditions chamber. The research outcomes showed that the YLD symptoms severity and APV1 titer abundancy were markedly higher in the plants maintained at low temperature (16/22 ± 2°C, night/day), as compared to high temperature (27/34 ± 2°C, night/day) and room temperature (24/26 ± 2°C, night/day), which reveals that temperature is the main factor that plays a key role in YLD symptoms severity and APV1 titer accumulation. It has been observed that higher temperature negatively affects viral replication. The symptoms inhibition at 34°C was associated with low viral titer accumulation. It was assumed that extremely high temperatures prohibit virus replication and movement, and similar results have been reported for potato leaf roll virus (PLRV) at high temperatures (Tamada and Harrison, 1981). It has been demonstrated that pathogen multiplication efficiency was also affected by the temperature at which the virus gets transmitted (Feil and Purcell, 2001), and the establishment of infection in the host (Chu and Volety, 1997). The coat protein (CP) of turnip mosaic virus (TuMV) accumulates abundantly inside the plants maintained at 23/28°C, while the symptoms were expressed at 18/28°C temperature as compared to plants maintained at 35°C (Chung et al., 2015). The expression of Barley yellow dwarf virus-PAV (BYDV-PAV) and symptoms severity of the BYDV-PAV-associated disease were significantly greater at elevated temperature (10.0/21.1°C, night/day) as compared to ambient temperature (Nancarrow et al., 2014).

## Conclusion

Previously, we observed anecdotally that under cold and dry weather conditions, the YLD symptoms severity becomes significantly high as compared to rainy and hot weather. Thus, based on the above observation, the present research work was conducted for the purpose of investigating the temperature and seasonal effects on YLD symptoms severity and APV1 titer accumulation both in the field under natural environmental conditions and in the laboratory under artificially controlled conditions, and it was concluded that symptoms severity was evidently higher in winter (December–February) as compared to autumn, i.e., rainy and moderate weather conditions (September), and summer, i.e., hot weather (May) in the field under natural environmental conditions. The qRT-PCR and ELISA results of field samples reveal that APV1 titer accumulates abundantly in cold and dry weather conditions than in rainy and hot weather conditions, and based on these assays, the YLD was closely associated with APV1. After confirmation of the seasonal effects on YLD symptoms and APV1 titer accumulation, we further conducted the same experiment under different temperatures, i.e., room, low, and high, in a chamber under controlled conditions for the purpose of evaluating the role of temperature in YLD intensity. Our results revealed that the YLD symptoms and APV1 titer accumulation were remarkably higher at low temperatures than at room and high temperatures. Thus, based on these findings, we concluded that temperature is one of the main factors that play a key role in YLD intensity. These findings represent empirical data about YLD symptoms emergence and APV1 accumulation that can be used to inform predictive models about the impact of different temperatures and seasons on YLD epidemiology.

## Data availability statement

The original contributions presented in the study are included in the article/supplementary materials, further inquiries can be directed to the corresponding author.

## Author contributions

XH conceived and designed the experiments; LK, RZ, and XC performed the experiments. XH and HT analyzed the data. LK wrote the paper. RZ, XC, ZX and XH revised the paper. All authors discussed the results and contributed to the final manuscript.

## Funding

This research work was financially supported by the Project of Sanya Yazhou Bay Science and Technology City, Grant No. SCKJ-JYRC-2022-71.

## Conflict of interest

The authors declare that the research was conducted in the absence of any commercial or financial relationships that could be construed as a potential conflict of interest.

## Publisher’s note

All claims expressed in this article are solely those of the authors and do not necessarily represent those of their affiliated organizations, or those of the publisher, the editors and the reviewers. Any product that may be evaluated in this article, or claim that may be made by its manufacturer, is not guaranteed or endorsed by the publisher.
